# Determination of the Mutagenicity Potential of Sankol Herbal Medicine by Single Cell Gel Electrophoresis in Rat Hepatocytes in Comparison With H_2_O_2_

**Published:** 2012-08-25

**Authors:** Heibatullah Kalantari, Mohsen Rezaei, Masoud Mahdavinia, Rashid Jahangirnejad, Golnaz Varnaseri

**Affiliations:** 1 School of Pharmacy, Medicinal plant Research Center, Ahvaz Jundishapur University of Medical Sciences, Ahvaz, IR Iran

**Keywords:** Toxicity Tests, Comet Assay, Herbal Medicine

## Abstract

**Background:**

The increasing use of herbal drugs and their ease of accessibility and availability have necessitated the use of mutagenicity test to analyze their toxicity and safety.

**Objectives:**

This study aimed to evaluate the genotoxicity of Sankol herbal medicine in DNA breakage of rat hepatocytes in comparison with H_2_O_2_ by single cell gel electrophoresis technique or comet assay.

**Materials and Methods:**

In the current study hepatocytes were prepared from male wistar rats. Hepatocytes cells were counted and kept in a bioreactor for 30 minutes,then cells were exposed to Sankol herbal medicine at doses of 100, 200 and 400 μl/ml. Buffer 4 (incubation buffer) and H_2_O_2_ were used for one hour as negative and positive control respectively. After 30 minutes cell suspension with low melting point agarose was put on precoated slides and covered with agarose gel. Then lysing, electrophoresis, neutralization and staining were carried out. Finally the slides were analyzed by fluorescence microscope. The parameter under this analysis was the type of migration which was determined according to Kobayashi pattern.

**Results:**

Results of the study indicated that by increasing the dose of Sankol herbal medicine, the DNA damage slightly increased (P < 0001).

**Conclusions:**

In overall compared to the positive control, significant differences were observed which indicated that the crude extract of Sankol in vitro did not have mutagenic effect.

## 1. Background

Fossils date back human use of plants as medicine to approximately 60000 years ago. Today, almost 65% of the world’s population rely on plants as an integral part of their primary health care. There have been many validations of traditional remedies through scientific research, andthe use of ethnomedical information has also contributed to health care world wide through the isolation of bioactive compounds for direct use in medicine. The adverse effects of widely used plants are not well documented in the literature. Based on their long-term use by man, one might expect the plants used in traditional medicine to have low toxicity. However, recent investigations have indicated that many plants used as food or in traditional medicine have mutagenic effects in in vitro assay which raises concerns about the potential mutagenic hazards resulting from the long-term use of medicinal plants (1, 2, 3, 4, 5, 6, 7, 8, 9).

In Iranian traditional medicine, Sankol is an example of plants that are widely used and, Sankol oral drop contains extracts of Foeniculum vulgare٫ Laurus nobilis٫ Tribulus terrestris٫ Cuminum cyminum٫ Cucumis melo٫ Zea mays٫ Cerasus avium which are widely used as an antispasmodic and remover of kidney and urinary tract stones. The active ingredients of medicinal plants in Sankol drop directly relax the smooth muscles of urinary tract and along with their diuretic activity remove the kidney stones up to 7 mm in diameter, and facilitate the expelling of kidney precipitations. Sankol Drop has a very potent anticholinergic activity and thus alleviates the spasm and colic pain of urinary tracts due to stone presence in kidney (10). Therefore, one of the objectives of the current study was to evaluate the safte activity of Sankol as a herbal medicine by in vitro method on hepatocytes.

The biological determination activities of in vitro methods for natural products have changed in recent years; one of the recent developments is comet assay, which gives a ratio between the viable cells in the cell culture to total cells in the culture. These techniques are considered rapid and economical to evaluate the mutagenicity or genotoxicity of compounds (11, 12).

## 2. Objectives

According to the potential therapeutic use of Sankol herbal medicine and the absence of data on its genetic toxicity in eukaryotes, the current study was conducted to evaluate the potential in vitro mutagenic effects in terms of DNA damage in rat hepatocytes by Single Cell Gel Electrophoresis technique.

## 3. Materials and Methods

The animal used in this experiment was a wistar rat (250 – 300 g weight) received from Razi Institute animal house, Iran. The rat was housed in a polyethylene cage in a climate controlled environment with a 12- hour (07.00 to 19.00) day length and ad libitum access to food and water. Hepatocyte extraction was prepared by IP injection of ketamin 90 mg/kg and xylazine 10 mg/kg for anesthesia. Then the rat was dissected and by IV injection of heparin the canulation of liver was made. Liver was washed with washing buffer for 10 minutes and then with perfusion buffer (colagenase buffer) for 12 minutes. The isolated liver was transferred in to a petri dish containing washing buffer to separate hepatocytes, then the cell suspension was filtered and the filtrate was centrifuged for 3 minutes at 1500 rpm. The upper layer was discarded and 10 ml of washing buffer was added to the lower layer, mixed well and then from the mixture 100 µl was mixed with 200 µl of incubation buffer and 300 µl of trypan blue to count the cells. A little amount of cell suspension was poured on Neubar slide and the cells were counted. Mean of the viable cells and dead cells were calculated as follows: % of viable cells = (mean of viable cells/total cells) × 100. Then hepatocytes were counted until the ratio of 106 cells was achived (13). A desired amount of the cell suspension was kept in a bioreactor bath at 37 C° atmosphere of 10 % O2, 85 % N2 and 5 % CO2 for 30 minutes and then transfered to five bioreactor flasks containing hepatocytes. Then the hepatocytes were exposed to Sankol herbal medicine (purchased from goldaru . co. Iran) for a period of 60 minutes at doses of 100, 200 and 400 µl/ml (test groups) respectively. Buffer 4 (which containd HEPES 0.75 g and Krebs 250 ml) was used as negative control and 10 µl/ml of H_2_O_2_ was used as positive control. Then from each bioreactor flask 1 ml of the hepatocyte cell suspension was taken and diluted to 10 ml with low melting point agarose and then 100 µl of this suspension was poured on precoated slides and then covered with cover slips. Slides were kept for 15 to 20 minutes in a horizontally placed iced tray till solidified. Next, the slides were uncovered and then kept in a lysing solution for one hour then removed and washed by D-ionized water, kept for 20 minutes in electrophorese buffer and electrophoresis was done at 25 V and 300 mA for 20 minutes , then washed three times by neutralized buffer each time for five minutes. Slides were immersed in ethidium bromide solution for five minutes ,according to the method described by Speit and Hartmann (14), which is based on the original work of Singh et al.(15) slides were analyzed by fluorescence microscope. The extent and distribution of DNA damage indicated by comet assay was evaluated by examining cells. The cells were visually scored into comet classes according to tail size class (16, 17, 18) Class 0 = no tail, Class 1 = tail shorter than the diameter of the head (nucleous) , Class 2 = tail length 1 to 2x the diameter of the head and Class 3 = tail longer than 2x the diameter of the head. Comet without head and those with nearly all the DNA in the tail or with a very wide tail, were excluded from the evaluation because they probably represented dead cells (19, 20) tail length and the mutagenic Index were calculated based on the following formula MI = (0NMC+ 1SMC+ 2MMC+ 3LMC) /200, or could be expressed as NMC = No migration cells (score 0), SMC = Short migration cells (score 1) ,MMC = Medium migration cells (score 2), LMC = Long migration cells (score 3).

## 4. Results

In Iran, like other countries, it is very common to use herbal medicine to treatdiseases; therefore, their safety features such as mutagenecity potential should be evaluated by techniques like single cell gel electrophoresis. This technique, is a sensitive, reliable, and important tool to evaluate the in vitro and in vivo genotoxic potential of bioactive compounds.

**Figure 1:**
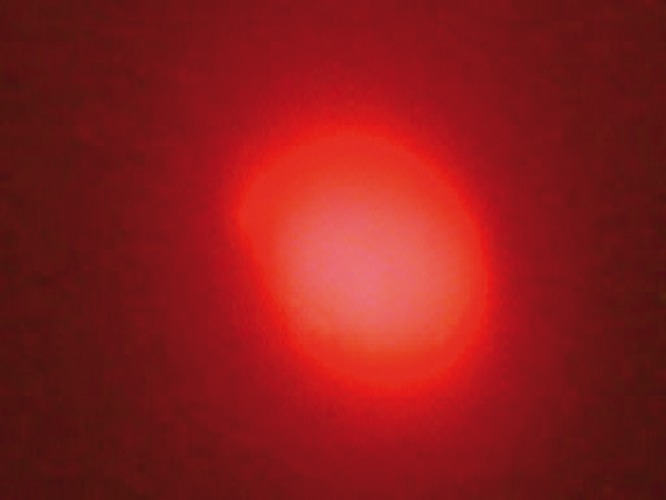
Microscopic Photograph 1 Score 0

**Figure 2:**
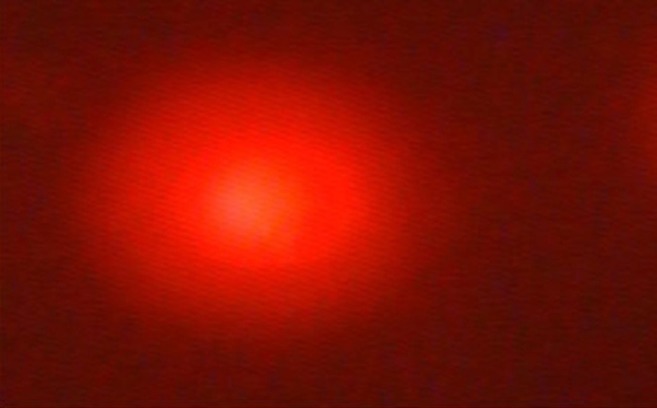
Microscopic Photograph 2 score 1

**Figure 3:**
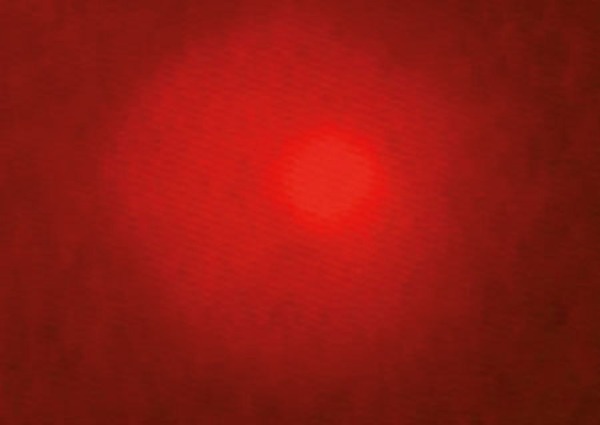
Microscopic Photograph 3 Score 2

**Figure 4:**
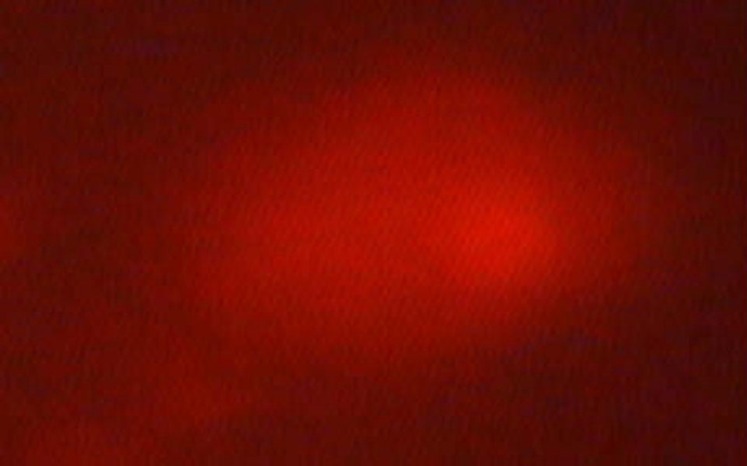
Microscopic Photograph 4 Score 3

The frequency percentage of rat hepatocytes comet pattern at different doses of Sankol herbal medicine and its comparison with positive control (H_2_O_2_) in 60 minutes after exposure which indicates significant differences P < 0.0001 is indicated in Table 1. Similarly, as the percentage of mutagenic index (MI) and damaged cells treated by Sankol herbal medicine at different doses, and its comparison with positive control (H_2_O_2_) in 60 minutes after exposure which indicates significant differences P < 0.0001,are indicated in Table 2. The microscopic Figures 1, 2, 3, 4 present the comet in scoring pattern from 0 score to score 3 which indicate the comet length. Comets from the broken ends of negatively charged DNA molecules became free and migrated towards the anode in the electric field. The assay provided direct determination of the extent of DNA damage in individual cells and the extent of DNA damage assessed from the length of DNA migration. As expected , positive and negative controls comparison indicated statistically significant difference.

**Table 1:** The percentage of rat hepatocytes comet pattern frequency at different doses of Sankol herbal medicine and its comparison with positive control (H_2_O_2_) in 60 minutes after exposure which indicates significant differences P < 0.0001.

Substance	Score 0	Score 1	Score 2	Score 3
H2O2 10 µl/ml	6 ± 0.4	16 ± 1.6	50 ± 1.03	28 ± 0.57
Sankol 0 µl/ml	94 ± 1.6	5.5 ± 1.09	0.5 ± 0.57	0 ± 0
Sankol 100 µl/ml	85 ± 1.22	13 ± 0.4	2 ± 0.81	0 ± 0
Sankol 200 µl/ml	80 ± 1.6	15 ± 0.4	5 ± 1.2	0 ± 0
Sankol 400 µl/ml	80 ± 1.7	13 ± 0.66	7 ± 1.15	0 ± 0

**Table 2:** The percentage of mutagenic index (MI) and damaged cells treated by Sankol herbal medicine at different doses and its comparison with positive control (H_2_O_2_) in 60 minutes after exposure which indicates significant differences P < 0.0001

Substance	MI	Damage cells ( Score 2 + Score 3)
H2O2 10 µl/ml	51 ± 1.03	78 ± 1.2
Sankol 0 µl/ml	1.6 ± 0.51	0.5 ± 0.57
Sankol 100 µl/ml	4.25 ± 0.98	2 ± 1.6
Sankol 200 µl/ml	6.25 ± 1.42	5 ± 2.3
Sankol 400 µl/ml	6.75 ± 1.73	7 ± 1.44

The results of the study indicated that the mutagenicity index of positive control was 51 % , the mutagenicity index for the lower dose of Sankol herbal medicine after 60 minutes was 4.25 %, and the mutagenicity index of higher dose of the Sankol herbal medicine after 60 minutes was 6.75 %. These findings indicated thatby the increase of dose the Sankol herbal medicine mutagenicity index increased but the increase was not significant ,compared with positive control P < 0.0001.

## 5. Discussion

Comparedto previous studies based on genotixicity study by comet assay the presentresults provided additional confirmation that high doses of Sankol herbal medicine did not cause significant DNA damage. As the herbal medicine contains antioxidant agents,they may protect the DNA damage by chemicals and prevent mutagenicity. In conclusion the results indicated that by increasing the dose of Sankol herbal medicine the DNA damage slightly increased but in overall , compared to the positive control ,significant differences were observed which indicated that the Sankol herbal medicine in vitro did not have mutagenic effect. 
